# NLRP3 inflammasome activation is involved in Ang II-induced kidney damage via mitochondrial dysfunction

**DOI:** 10.18632/oncotarget.11091

**Published:** 2016-08-05

**Authors:** Yi Wen, Yiran Liu, Taotao Tang, Linli Lv, Hong Liu, Kunling Ma, Bicheng Liu

**Affiliations:** ^1^ Institute of Nephrology, Zhong Da Hospital, Southeast University, Nanjing, Jiangsu, China

**Keywords:** NLRP3 inflammasome, angiotensin II, mitochondrial dysfunction, RAS, Pathology Section

## Abstract

Growing evidence has shown that NLRP3 inflammasome activation promotes the development of tubulointerstitial inflammation and progression of renal injury. We previously found that mitochondrial dysfunction is a critical determinant for the activation of NLRP3 inflammasome in albumin-overload rats. Angiotensin (Ang) II plays an important role in mitochondrial homeostasis. Here, we investigated the role of Ang II in NLRP3 inflammasome activation and the involvement of mitochondrial dysfunction in this process. *In vitro*, Ang II triggered NLRP3 inflammasome activation in a dose- and time-dependent manner, and this effect is mediated by AT1 receptor rather than AT2 receptor. MitoTEMPO, a mitochondrial targeted antioxidant, attenuated Ang II induced mitochondrial reactive oxygen species (mROS) production and NLRP3 inflammation activation. Following chronic Ang II infusion for 28 days, we observed remarkable tubular epithelial cells (TECs) injury, mitochondrial damage, and albuminuria in WT mice. However, these abnormalities were significantly attenuated in AT1 receptor KO mice. Then, we examined the role of mitochondria in Ang II-infused mice with or without mitoTEMPO treatment. As expected, Ang II-induced mitochondrial dysfunction and NLRP3 inflammasome activation was markedly inhibited by mitoTEMPO. Notably, NLRP3 deletion signally protected TECs from Ang II-triggered mitochondrial dysfunction and NLRP3 inflammasome activation. Taken together, these data demonstrate that Ang II induces NLRP3 inflammasome activation in TECs which is mediated by mitochondrial dysfunction.

## INTRODUCTION

Activation of the renin-angiotensin system (RAS) increases blood pressure, which leads to progressive kidney injury. Both type 1 angiotensin receptor (AT1R) and type 1 angiotensin receptor (AT2R) are important receptors of the renin-angiotensin system. The activation of AT1R by the principal product of the RAS, angiotensin (Ang) II, maintains blood pressure and fluid homeostasis, while the capacity of AT2R remains elusive [1]. It is demonstrated that AT1R and AT2R have opposite functions during physiological and pathological conditions. The activation of AT1R facilitates proliferation of vascular endothelial cells and mediates angiogenesis, while AT2R is characterized by anti-proliferation effect [2]. Previous studies showed that protection of renal function provided by AT1 receptor blockers (ARBs) cannot be explained by decreased blood pressure alone [3, 4], and this blood pressure-independent protection of ARBs is mediated by the inhibition of Ang II-induced inflammatory response. In Ang II-infusion models, Ang II promotes kidney injury by causing infiltration of monocytes/macrophages and T lymphocytes [5, 6]. *In vitro* studies indicated that Ang II stimulates cell proliferation, NF-κB activation, and generation of pro-inflammatory cytokines [7, 8]. These results suggest that Ang II-induced inflammatory reactions play an important role in the development and progression of renal damage. However, the exact mechanism of Ang II-induced inflammation remains to be clarified.

The Nod-like receptors are intracellular sensors of danger-associated molecular patterns (DAMPs) and pathogen-associated molecular patterns (PAMPs). As an important member of the NLR family, NLRP3 is constituted of a leucine-rich repeat (LRR) region, a conserved nucleotide-binding oligomerization domain (NOD) and a N-terminal effector domain [9]. Previous studies showed that NLRP3 could be activated by a wide range of danger signals, such as mitochondrial dysfunction, extracellular ATP and damaged nucleic acids. The activation of NLRP3 leads to the formation of NLRP3 inflammasome, a macromolecular complex consisted by the adaptor part apoptosis-associated speck-like protein containing a CARD (ASC) and the effector protease caspase-1, to promote the maturation and secretion of IL-1β and IL-18 [10]. Recent studies indicated that NLRP3 inflammasome and downstream cytokines are widely expressed and contribute to inflammatory diseases, including atherosclerosis, gouty arthritis and type 2 diabetes [11–14]. Our previous studies showed a causal relationship between albuminuria and NLRP3 activation in BSA-overload rats, which is mediated by mitochondrial dysfunction [15]. However, the role of NLRP3 inflammasome activation in Ang II-induced renal injury has not been defined.

Mitochondria are important intracellular organelles which are responsible for energy supply via oxidative phosphorylation. In pathological conditions, mitochondrial damage increases ROS production, and the mROS accumulation results in mitochondrial DNA damage and progressive respiratory chain dysfunction, which eventually leads to mitochondrial dysfunction [16]. We previously found that mitochondrial dysfunction was induced by albumin overload and contributes to NLRP3 inflammasome activation. However, the role of mitochondrial dysfunction in Ang II-induced inflammation remains speculative. The purpose of this study was to investigate whether Ang II stimulates NLRP3 inflammasome activation through mitochondrial dysfunction during kidney damage.

## RESULTS

### Ang II stimulated NLRP3 inflammasome activation *in vitro*

**Figure 1:**
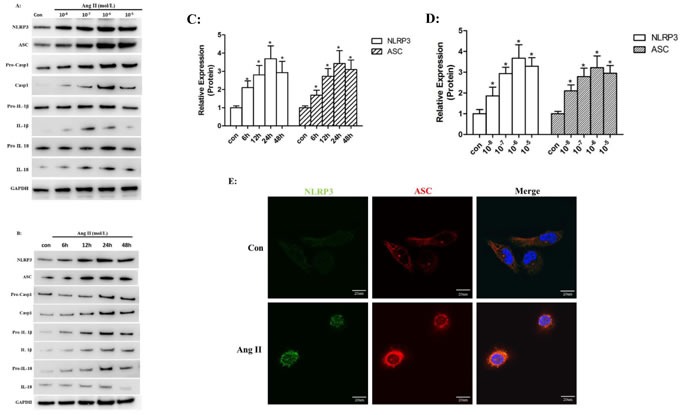
Ang II stimulated NLRP3 inflammasome activation *in vitro* **A**-**B**: Western Blot analysis of the NLRP3, ASC, pro-casp1, pro-IL-1β, and pro-IL-18 (cell lysates) and processed casp1, IL-1β, and IL-18 (supernatant) in HK-2 cells after stimulated by Ang II in different dose- (10^−8^, 10^−7^, 10^−6^, and 10^−5^ mol/L) for different time- (6, 12, 24, and 48 hours of incubation). **C**-**D**: Quantification of the protein expression levels. The data are presented as the means ± SD from three independent experiments. *P<0.05 vs. the control group. **E**: The NLRP3 (green) and ASC (red) protein distribution after Ang II (10^−6^ mol/L) treatment for 24 h was localized by confocal microscopy

To investigate the role of Ang II in NLRP3 inflammasome activation, we stimulated HK-2 cells with different doses of Ang II over a range of time periods. We found that protein levels of NLRP3 and ASC were significantly increased by Ang II stimulation in a time- (6, 12, 24, and 48 hours of incubation) and dose- (10^−8^, 10^−7^, 10^−6^, and 10^−5^ mol/L) dependent manner which peaks at 24 h with 10^−6^ mol/L Ang II (Figure 1A-D). Similarly, we detected increased expression of mature caspase-1, IL-1β and IL-18 after Ang II stimulation. We then examined NLRP3 and ASC expression in HK-2 cells by immunofluorescence and found that NLRP3 and ASC were overproduced and co-localized around the nucleus after Ang II stimulation (Figure 1E).

### Ang II triggered maturation of IL-1β and IL-18 via AT1R rather than AT2R

**Figure 2:**
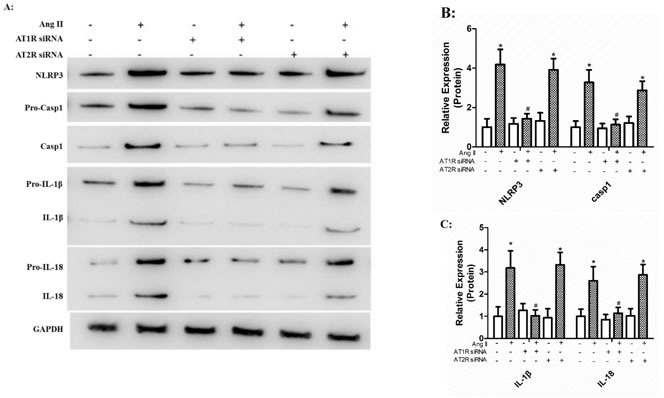
Ang II triggered maturation of IL-1β and IL-18 via AT1R rather than AT2R **A**: The levels of NLRP3, ASC, pro-casp1, pro-IL-1β, and pro-IL-18 (cell lysates) and processed caspase-1, IL-1β, and IL-18 (supernatant) in HK-2 cells transfected with siRNA for AT1R or AT2R prior to Ang II stimulation were detected by Western Blotting. **B**-**C**: Quantification of the protein expression levels. The data are presented as the means ± SD from three independent experiments. *P<0.05 vs. the control group. #P<0.05 vs. the Ang II stimulation group.

To examine the exact role of AT1R and AT2R in Ang II-induced NLRP3 inflammasome activation, HK-2 cells were transfected with siRNA for AT1R or AT2R 24 h prior to Ang II treatment (10^−6^ mol/L). We found that AT1R siRNA significantly reduced Ang II-induced NLRP3 overexpression and maturation of IL-1β and IL-18 (Figure 2A). However, HK-2 cells transfected with AT2R siRNA showed robust expression of IL-1β and IL-18 in response to Ang II stimulation (Figure 2A-C).

### Ang II induced mitochondrial dysfunction and mROS overproduction *in vitro*

**Figure 3:**
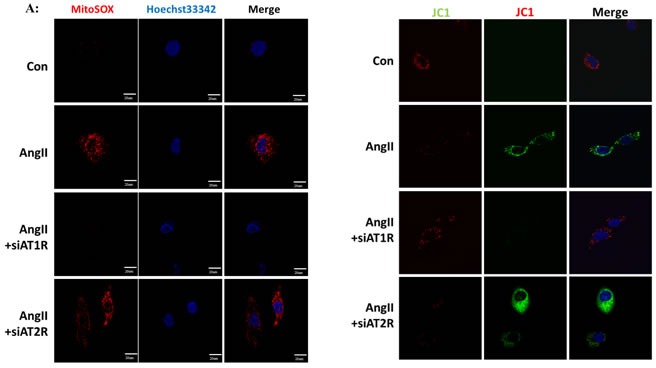
Ang II induced mitochondrial dysfunction and mROS overproduction *in vitro* **A**: Confocal microscopy analysis of mitochondria-generated ROS by MitoSOX staining in living HK-2 cells pre-treated with siAT1R or siAT2R before Ang II stimulation. **B**: Confocal microscopy analysis of mitochondrial transmembrane potential (MMP) by JC-1 staining in living HK-2 cells pre-treated with siAT1R or siAT2R before Ang II stimulation.

We then performed *in vitro* studies to investigate the role of Ang II in mitochondrial dysfunction. The ROS production was detected by immunofluorescence using mitoSOX, which was widely used for oxidative stress measurement. As shown in Figure 3A, Ang II induced mitochondrial ROS overproduction as compared with the controls. In accordance with Western Blot analysis, AT1R siRNA rather than AT2R siRNA attenuated Ang II-induced mROS overproduction (Figure 3A). JC-1 staining was applied to evaluate the mitochondrial membrane potential (MMP) of HK-2 cells. We observed that Ang II significantly increased green fluorescent levels and inhibited red fluorescent levels, which indicated the decline of MMP. Similarly, AT1R siRNA rather than AT2R siRNA inhibited Ang II-induced MMP decline (Figure 3B).

### MitoTEMPO scavenged mROS to inhibit NLRP3 inflammasome activation

**Figure 4:**
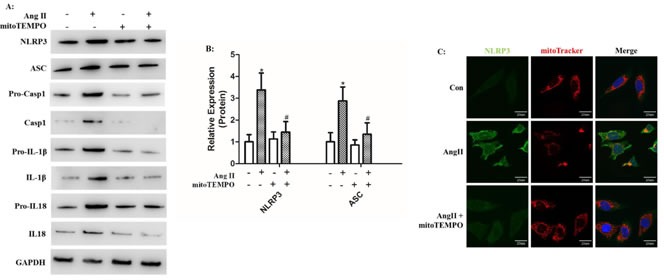

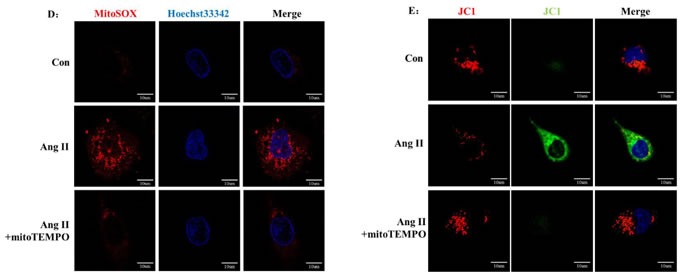
MitoTEMPO scavenged mROS to inhibit NLRP3 inflammasome activation **A**: HK-2 cells were pre-incubated or not pre-incubated with MitoTEMPO and then treated with Ang II. The levels of NLRP3, ASC, pro-casp-1, pro-IL-1β, and pro-IL-18 expression in the cell lysates and caspase-1, IL-1β, and IL-18 expression in the supernatants were determined by Western Blotting. **B**: Quantification of NLRP3 and ASC protein expression levels. The data are presented as the means ± SD from three independent experiments. *P<0.05 vs. the control group. #P<0.05 vs. the Ang II stimulation group. **C**: The co-localization of NLRP3 (green) with the mitochondria (Mitotracker Red) were analyzed by confocal microscopy observation. **D**: Confocal microscopy analysis of mitochondria-generated ROS by MitoSOX staining in living HK-2 cells treated with mitoTEMPO. **E**: Confocal microscopy analysis of mitochondrial transmembrane potential (MMP) by JC-1 staining in living HK-2 cells treated with mitoTEMPO.

Because mROS was proposed to be involved in NLRP3 inflammasome activation, we treated HK-2 cells with MitoTEMPO, a mitochondria-targeted antioxidant, during Ang II stimulation. Western Blot analysis showed that the secretion and maturation of IL-1β and IL-18 was significantly prevented by MitoTEMPO treatment (Figure 4A-B). We then stained mitochondria by MitoTracker to examine the co-localization of NLRP3 and mitochondria *in vitro*. In the resting state, NLRP3 proteins were widely distributed in the cytoplasm in granular structures, and the mitochondria had a filamentous morphology. Ang II stimulation changed the mitochondria into fragmented morphology and increased the combination with NLRP3 surrounding the nucleus. The application of mitoTEMPO inhibited NLRP3 upregulation and co-localization with fragmented mitochondria (Figure 4C). We also found that MitoTEMPO treatment significantly attenuated Ang II-induced mROS overproduction and MMP decline (Figure 4D-E).

### Biochemical and pathological changes of Ang II-induced kidney damage *in vivo*

**Figure 5:**
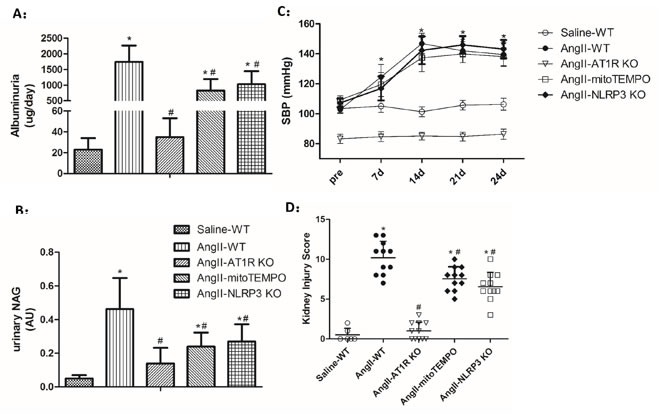
Biochemical and pathological changes of Ang II-induced kidney damage *in vivo* **A**-**B**: Quantification of albuminuria and NAG levels. The data are presented as the means ± SD from three independent experiments. **C**: Time course of the SBP levels. **D**: Tubular injury scores obtained for each group. The data are the mean scores (*P<0.05 vs. the saline-WT group. #P<0.05 vs. the Ang II-WT group).

To further test the effect of Ang II on the initiation and progression of renal injury, different types of uninephrectomized mice were infused with Ang II, and WT mice infused with saline were taken as the control group. After 4 weeks of Ang II infusion, urinary levels of albumin and N-acetyl-β-D-glucosaminidase (NAG) were increased in WT, mitoTEMPO-treated and NLRP3 KO mice compared to the WT controls (Figure 5A-B). However, Ang II-infused mitoTEMPO-treated and NLRP3 KO mice showed much lower albuminuria and NAG levels than Ang II-infused WT mice. In addition, both albuminuria and NAG levels were significantly reduced in AT1R KO mice (Figure 5A-B). Following chronic infusion of Ang II for 4 weeks, the blood pressure (SBP) was measured by the tail-cuff method. We found that Ang II induced significant and similar increase of SBP in mitoTEMPO-treated and NLRP3 KO mice compared to the WT controls, demonstrating the hypertension-independent protection of mitoTEMPO and NLRP3 deletion (Figure 5C). However, Ang II-infused AT1R KO mice showed much lower blood pressure levels than the WT controls, indicating the important role of AT1Rs in blood pressure maintenance (Figure 5D).

### AT1R deletion attenuated Ang II-induced NLRP3 inflammasome activation *in vivo*

**Figure 6:**
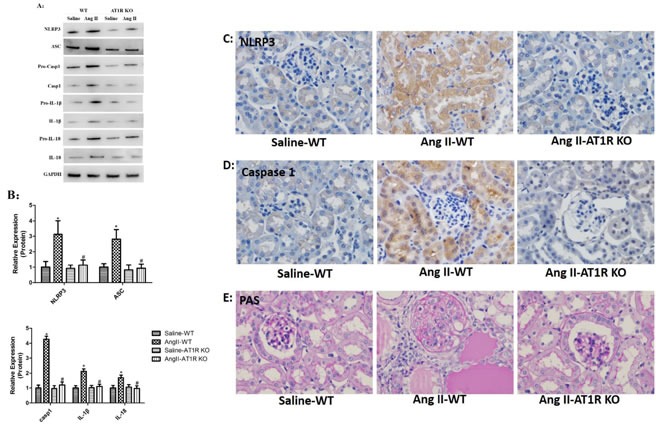
AT1R deletion attenuated Ang II-induced NLRP3 inflammasome activation *in vivo* **A**: NLRP3, ASC, casp-1, IL-1β, and IL-18 expression in kidneys were determined by Western Blotting. **B**: Quantification of the expression levels of these proteins. The data are the means ± S.D. (*P<0.05 vs. the saline-WT group and #*P*<0.05 vs. the Ang II-WT group). **C**-**D**: Immunohistochemistry analysis of NLRP3, and caspase-1 expression and localization in kidney tissue. **E**: PAS staining of saline-WT, Ang II-infused WT and AT1R KO mice

To examine the activation of NLRP3 inflammasome *in vivo*, the protein levels of NLRP3, ASC, caspase-1, IL-1β and IL-18 in tubular epithelial cells were evaluated by Western Blot analysis. We observed that Ang II infusion markedly triggered NLRP3 inflammasome activation in WT mice. Consistent with *in vitro* study, the NLRP3 inflammasome activation induced by Ang II was blocked by AT1R deletion (Figure 6A-B). In addition, the immunohistochemical staining showed that NLRP3 and caspase-1 were significantly increased and expressed in tubular epithelial cells of Ang II-infused WT mice, and these changes were prevented in AT1R KO mice (Figure 6C-D). Consistent with previous results, we observed marked tubular interstitial damage in Ang II-infused WT mice, such as extracellular matrix overproduction, increased inflammatory cell infiltration, protein cast formation and tubular dilatation. And these Ang II-induced kidney injures were significantly attenuated by AT1R deletion (Figure 6E).

### AT1R deletion attenuated Ang II-induced mitochondrial dysfunction *in vivo*

**Figure 7:**
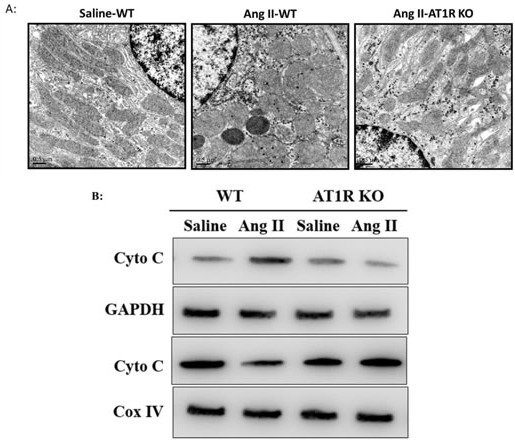
AT1R deletion attenuated Ang II-induced mitochondrial dysfunction *in vivo* **A**: The ultrastructure of tubular epithelial cells in saline-treated and Ang II-infused mice was observed by TEM. **B**: Western Blot analysis of the release of cytochrome c from mitochondria to cytoplasm.

We further examined the ultrastructural alterations of mitochondria in tubular epithelial cells after Ang II infusion. The ultrastructural features of mitochondria were linear in control group. In contrast, the mitochondria in Ang II-infused WT mice displayed balloon-like morphological changes with edema, abundant matrix, and swelled cristae (Figure 7A). Additionally, Western Blot analysis showed that cytochrome C was released from the mitochondria to the cytosol in Ang II-infused WT mice (Figure 7B). However, the ultrastructural and functional changes of the mitochondria triggered by Ang II were negligible in AT1R KO mice (Figure 7B).

### MitoTEMPO attenuated Ang II-induced NLRP3 inflammasome activation and renal damage

**Figure 8:**
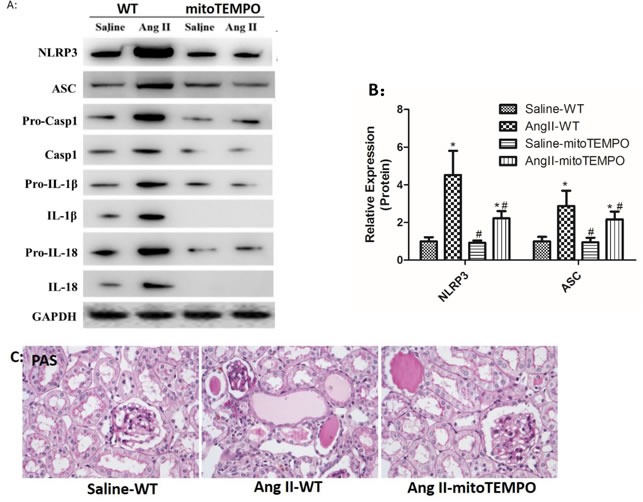
MitoTEMPO attenuated Ang II-induced NLRP3 inflammasome activation and renal damage **A**: NLRP3, ASC, casp-1, IL-1β, and IL-18 expression in kidneys were determined by Western Blotting. **B**: Quantification of the expression levels of these proteins. **C**: PAS staining of saline-WT, Ang II-infused WT and mitoTEMPO-treated mice. The data are the means ± S.D. (*P<0.05 vs. the saline-WT group and #*P*<0.05 vs. the Ang II-WT group).

Consistent with *in vitro* studies, mitoTEMPO treatment notably inhibited Ang II induced NLRP3 inflammasome activation *in vivo*. Western Blot analysis indicated that maturation and secretion of IL-1β and IL-18 in Ang II-infused WT mice was significantly attenuated by mitoTEMPO treatment (Figure 8A-B). We also found that Ang II-infused WT mice showed significant inflammatory cell infiltration, protein cast formation and tubular dilatation, and these changes were markedly prevented by mitoTEMPO treatment (Figure 8C).

### NLRP3 deletion inhibited NLRP3 inflammasome activation and maintains mitochondrial homeostasis

**Figure 9:**
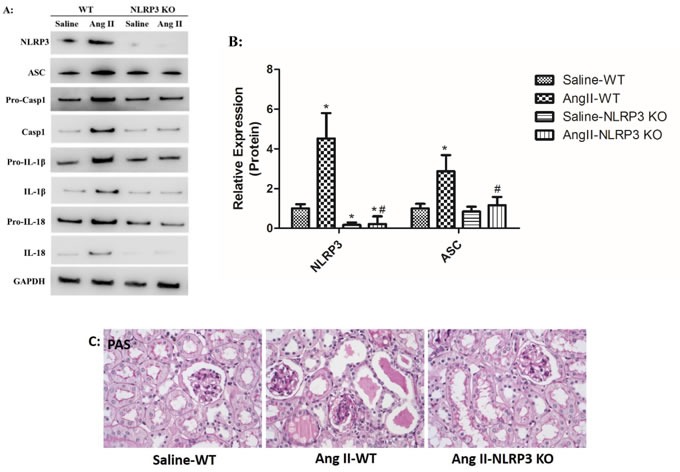

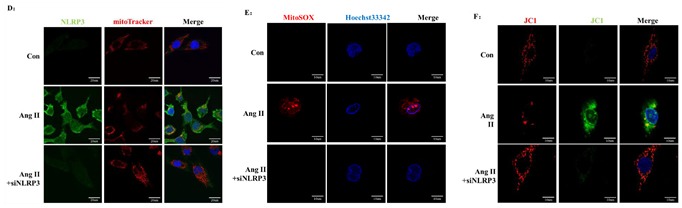
NLRP3 deletion inhibited NLRP3 inflammasome activation and maintains mitochondrial homeostasis **A**: NLRP3, ASC, casp-1, IL-1β, and IL-18 expression in kidneys were determined by Western Blotting. B: Quantification of the expression levels of these proteins. The data are the means ± S.D. (*P<0.05 vs. the saline-WT group and #*P*<0.05 vs. the Ang II-WT group). **C**: PAS staining of saline-WT, Ang II-infused WT and NLRP3 KO mice. **D**: The co-localization of NLRP3 (green) with the mitochondria (Mitotracker Red) were analyzed by confocal microscopy observation. **E**: Confocal microscopy analysis of mitochondria-generated ROS by MitoSOX staining in living HK-2 cells pre-treated with siAT1R. **F**: Confocal microscopy analysis of mitochondrial transmembrane potential (MMP) by JC-1 staining in living HK-2 cells pre-treated with siAT1R.

Ang II infusion triggered NLRP3 overproduction and NLRP3 inflammasome activation. *In vivo* studies showed that secretion of cleaved IL-1β and IL-18 was notably inhibited in Ang II-infused NLRP3 KO mice compared to Ang II-infused WT mice (Figure 9A-B). We also found that Ang II-induced kidney injury was markedly prevented by mitoTEMPO treatment (Figure 9C). The role of NLRP3 inflammasome in Ang II-induced mitochondrial dysfunction was also investigated. *In vitro* studies showed that NLRP3 silence rescued fragmented mitochondria from combination with NLRP3 after Ang II stimulation (Figure 9D). In addition, Ang II-induced mROS overproduction and MMP decline was significantly reversed by NLRP3 silence (Figure 9E-F). These results strongly suggested the detrimental role of NLRP3 in Ang II-induced mitochondrial dysfunction and kidney damage.

## DISCUSSION

RAS activation is involved in various kinds of kidney disease, such as focal segmental glomerulosclerosis (FSGS), diabetic nephropathy, lupus nephritis and hypertensive nephropathy [17]. It is reported that Ang II was the major influencing factor of RAS and contributed to the development of hypertension and organ damage. However, clinical trials demonstrated that the blockage of organ damage by RAS cannot be explained by blood pressure decline alone. Recent studies demonstrated that Ang II stimulated various pro-inflammation responses, including macrophage/lymphocyte proliferation, NF-κB activation, and chemokine production [7, 8]. However, an important unanswered question in understanding Ang II-induced renal inflammation is the mechanism of tubular epithelial injury directly influenced by Ang II. Our results indicate that mitochondrial reactive oxygen species overproduction after Ang II stimulation leads to NLRP3 inflammasome activation which contributes to renal inflammation and damage.

NLRP3 is a cytosolic receptor that binds to different ligands resulting in the production of pro-inflammatory cytokines. Recent studies suggested that NLRP3 could be activated by various danger signals to promote inflammation [18]. Vilaysane et al. also demonstrated that NLRP3 inflammasome was involved in unilateral ureteral obstruction (UUO)-induced renal inflammation and contributed to the progression of chronic kidney disease [12]. Recently, it is also reported that CP-456,773, an NLRP3-specific inflammasome inhibitor, strongly attenuated crystal-induced renal inflammation and fibrosis [19]. Our previous study showed that endoplasmic reticulum stress was involved in NLRP3 inflammasome activation during Ang II-induced HK-2 cells injury. Among the subtypes of Ang II receptors, AT1 receptor and AT2 receptors are the most important ones. However, the exact role of these two receptors in Ang II-induced NLRP3 inflammasome activation remains unclear. Here we characterized the NLRP3 inflammasome activation both *in vitro* and *in vivo* by evaluating the protein levels of NLRP3 inflammasome members. Through this analysis, we found that NLRP3 inflammasome was activated by Ang II in a time- and dose-dependent manner. The NLRP3 inflammasome activation was greatly inhibited when we applied HK-2 cells with AT1R siRNA before Ang II stimulation *in vitro*. However, silence of AT2R did not affect the secretion of IL-1β and IL-18 after Ang II-stimulation. Consistent with this, the NLRP3 inflammasome activation was much lower in AT1R KO mice than WT mice after 4 weeks of Ang II infusion. In addition, the albuminuria and urinary NAG levels were also attenuated by AT1R deletion. PAS staining showed that Ang II-induced protein casts and inflammatory cells infiltration were significantly inhibited in AT1R KO mice. These data demonstrated that Ang II stimulated NLRP3 inflammasome activation mainly through AT1R rather than AT2R.

Recent studies reported that mitochondrial dysfunction is an important activator of NLRP3 inflammasome [20–23]. Zhou et al observed increased co-localization of NLRP3 and mitochondria in macrophages, indicating the essential role of mitochondria in NLRP3 inflammasome activation [20]. We previously demonstrated that the albumin-induced NLRP3 inflammasome activation was mediated by mitochondrial dysfunction, especially mitochondrial-originated ROS overproduction, in tubular epithelial cells. [15]. Haugen et al also found that Ang II triggered oxidative stress and heme oxygenase activation in renal proximal tubules [24]. Kim et al found that Ang II stimulated the generation of mitochondrial Nox4 and the overproduction of superoxide and hydrogen peroxide [25]. However, the exact role of mitochondrial dysfunction in Ang II-induced NLRP3 inflammasome activation remains unclear. Consistent with these findings, our study showed that the mitochondrial function was significantly damaged by Ang II *in vitro*, as demonstrated by mitochondrial ROS overproduction and membrane potential decline. In addition, application of MitoTEMPO, a mitochondria-targeted ROS scavenger, significantly attenuated mitochondrial dysfunction and NLRP3 inflammasome activation. These data strongly suggested an *in vitro* role of mitochondrial dysfunction in mediating Ang II-induced NLRP3 inflammasome activation. We further treated Ang II-infused WT mice with mitoTEMPO to examine its protective effect *in vivo*. In agreement with *in vitro* results, *in vivo* studies showed a protective role of mitoTEMPO in opposing Ang II-induced NLRP3 inflammasome activation. Meanwhile, the albuminuria, urinary NAG excretion, and renal tissue damage were ameliorated in mitoTEMPO-treated WT mice. These observations suggested that mitochondrial dysfunction was a crucial participant in Ang II-induced NLRP3 inflammasome activation.

In addition, we found that NLRP3 siRNA effectively attenuated mitochondrial dysfunction in HK-2 cells stimulated by Ang II, indicating that NLRP3 inflammasome activation might contribute to mitochondrial damage through unknown pathways. *In vivo* results demonstrated that NLRP3 deletion blocked Ang II-induced inflammasome activation and ameliorated Ang II-induced renal injury. We also found that blood pressure of NLRP3 KO mice was significantly increased by Ang II infusion. These data suggested that the NLRP3 inflammasome mediated Ang II-induced renal inflammation in a blood pressure-independent manner.

In conclusion, we first examined NLRP3 inflammasome activation in different types of mice infused with Ang II. Both *in vivo* and *in vitro* results strongly indicated that mitochondrial dysfunction contributed to NLRP inflammasome activation and was triggered by Ang II via AT1R. Based on the importance of NLRP3 inflammasome activation in the development and progression of renal damage, our novel findings emphasized the importance of clinical RAS blocking therapy. Targeting mitochondria and/or NLRP3 inflammasome may be a novel therapeutic strategy for the treatment of RAS activation-related kidney disease.

## MATERIALS AND METHODS

### Animals

Agtr1a KO and NLRP3 KO mice on the C57BL/6 background were purchased from Jackson laboratory and were backcrossed to the 129/SvEv mouse strain for ≥6 generations to increase their susceptibility to kidney damage[26]. Thereafter, heterozygotes on the 129/SvEv background were intercrossed to yield knock-out (KO) and wild-type (WT) littermates for our experiments. Eight- to 12-week-old male mice were used for the experiments. All experiments described in this manuscript were included in an animal use protocol approved by the Ethics Review Committees for Animal Experimentation of Southeast University.

### Ang II-infusion model

WT, Agtr1a KO, and NLRP3 KO mice on the 129/SvEv background underwent left nephrectomy, following which the mice were not experimented upon for 7 days to allow for wound healing and reestablishment of blood pressure rhythm. Then, the mice were continuously infused with Ang II (1000 ng·kg^−1^·day^−1^; Sigma; n≥11 mice per group) or saline (n=6 mice per group) using subcutaneous osmotic mini-pumps (ALZET model 2004) for 28 days, as previously described. MitoTEMPO was intraperitoneally administered at 0.5 mg/Kg twice a day for 4 weeks while WT mice continued Ang II-infusion. Blood pressure measurements were recorded using the tail-cuff method at baseline and during the 4 weeks of saline or Ang II infusion. On every weekend, the mice were placed in metabolic cages to collect urine for 24 hours.

### Urine and blood measurements

At the end of the 4-week infusion period, blood samples were collected from the heart, and the heart and kidney were harvested and weighed. Serum creatinine was quantified using the colorimetric assay kit (Roche Diagnostics). The concentrations of the proteins and albumin were measured in the collected urinary samples using specific ELISA kits (Jiancheng, Nanjing) according to the manufacturer's instructions. Albumin excretion is presented as micrograms of albumin per milligram of creatinine.

### Renal histological preparation and analysis

The right kidneys of the mice were harvested and weighed after perfusion with 50 ml saline (4°C). Portions of the renal cortex were fixed with 10% formalin and embedded in paraffin for periodic acid-Schiff (PAS) assay and immunohistochemistry. The remaining tissue was stored in liquid nitrogen for Western blotting.

### Immunohistochemical staining

Immunohistochemical staining was used to examine the expression and localization of the proteins of interest. Paraffin-embedded sections of the renal cortex were incubated with primary antibodies against NLRP3 (Adipogen, USA) and caspase-1 (Santa Cruz Biotechnology, USA).

The sections were then treated with immunohistochemistry kits (Maxim, China) matched to the species of the primary antibody. A semi-quantitative immunohistochemistry analysis was conducted by a pathologist using the Image Pro Plus image analysis system.

### Electron microscopy analysis

Renal cortical tissues were fixed in a mixture of 2.5% glutaraldehyde and 4% paraformaldehyde for transmission electron microscopy (TEM) examination of ultrastructural structure. After fixation and dehydration with ethanol, the samples were embedded in Durcupan resin for ultrathin-sectioning and ultramicroscopic observation.

### Cell culture and siRNA treatment

The HK-2 (human kidney 2) cell line was obtained from ATCC, and HK-2 cells were cultured in complete growth medium (Keratinocyte Serum Free Medium [GIBCO, USA]; Bovine Pituitary Extract [0.05 mg/ml, SCIENCELL, USA]; Human Recombinant Epidermal Growth Factor [5 ng/ml, GIBCO, USA]). The siRNA and transfection reagents were purchased from Invitrogen, and nonspecific scramble siRNA was used as a negative control. Transfection of HK-2 cells was performed in strict accordance with the manufacturer's protocol (Invitrogen, USA).

### Western blotting assays

Cell lysis solution and culture supernatants were collected for Western blot analysis as previously described [27]. Following the instructions of the total protein extraction kit (KeyGEN, Nanjing), whole-cell lysates were obtained from HK-2 cells and cortical tissues. The cell culture supernatants were precipitated using methanol and chloroform. Then, they were centrifuged, and the upper phase was discarded. The interphase was mixed with methanol, centrifuged for precipitation, and dried at 55°C. The protein pellets were resuspended in Laemmli buffer for sample preparation. Then, the proteins were separated by SDS-PAGE and transferred to PVDF membranes (Millipore, USA). After blocking the PVDF membranes with 5% serum for a half hour, they were incubated with primary antibodies (anti-NLRP3 [Adipogen, USA], anti-ASC, caspase-1, IL-1β, and IL-18 [Abcam, USA]) overnight at 4°C. Then, the membranes were washed and were incubated with secondary horseradish peroxidase-conjugated antibodies for 2 hours at room temperature, and the antibody-bound proteins were detected using the ECL advanced system (GE Healthcare, UK).

### Confocal microscopy visualization

HK-2 cells were cultured in confocal dishes 2 d before Ang II stimulation and were then subjected to MitoTracker Red staining (Gibco, USA). After 3 × 10 min of washing with PBS, the HK-2 cells were fixed with 4% PFA at 37°C for 15 min and then washed with PBS. The HK-2 cells were treated with Triton X-100 and blocked with 10% BSA in PBS. Then, they were incubated with primary antibodies overnight at 4°C. The HK-2 cells were washed with PBS, incubated with fluorescein-labeled secondary antibodies (Invitrogen, USA) and then rinsed with PBS again. Finally, nuclei were stained with DAPI (Invitrogen, USA). Additionally, living HK-2 cells in the different treatment groups were stained with MitoSOX (Gibco, USA) and washed with HBSS. After Hoechst 33342 staining of nuclei, the HK-2 cells were immediately visualized under a confocal microscope (Olympus FV 1000 Viewer).

In addition, HK-2 cells were prepared for quantification of MMP using the JC-1 kit (KeyGEN, China). Uptake of the fluorescent dye JC-1 from the cytoplasm to the mitochondria was observed by fluorescence microscopy and was measured using Image Pro-Plus software.

### Statistical analysis

All data are expressed as the means ± SD and were analyzed by one-way ANOVA using SPSS 20.0 statistical software. Nonparametric data were analyzed using the Mann–Whitney U test. P values <0.05 were considered statistically significant.
